# Signal enhancement ratio of multi-phase contrast-enhanced MRI: an imaging biomarker for survival in pancreatic adenocarcinoma

**DOI:** 10.1007/s00330-024-10746-z

**Published:** 2024-05-15

**Authors:** Cong Xia, Jin-rong Qu, Yi-ping Jiao, Chun-qiang Lu, Ben Zhao, Rong-jun Ge, Yue Qiu, Bu-yue Cao, Qian Yu, Tian-yi Xia, Xiang-pan Meng, Yang Song, Li-hua Zhang, Xue-ying Long, Jing Ye, Zhi-min Ding, Wu Cai, Sheng-hong Ju

**Affiliations:** 1 Cultivation and Construction Site of the State Key Laboratory of Intelligent Imaging and Interventional Medicine, Department of Radiology, Zhongda Hospital, Medical School of Southeast University, 87 Dingjiaqiao Road, 210009 Nanjing, Jiangsu China; 2 Department of Radiology, The Affiliated Cancer Hospital of Zhengzhou University, Henan Cancer Hospital, Zhengzhou, Henan China; 3 Institute for AI in Medicine, School of Artificial Intelligence, Nanjing University of Information Science and Technology, Nanjing, China; 4 School of Instrument Science and Engineering, Southeast University, Nanjing, China; 5 MR Scientific Marketing, Siemens Healthineers Ltd., Shanghai, China; 6 Department of Pathology, Zhongda Hospital, School of Medicine, Southeast University, Nanjing, China; 7 Department of Radiology, The Xiangya Hospital of Central South University, Changsha, China; 8 Department of Medical Imaging, Subei People’s Hospital, Medical School of Yangzhou University, Yangzhou, China; 9 Department of Radiology, Yijishan Hospital of Wannan Medical College, Wuhu, China; 10 Department of Radiology, The Second Affiliated Hospital of Soochow University, Suzhou, China

**Keywords:** Pancreatic adenocarcinoma, Multi-phase contrast-enhanced MRI, Signal enhancement ratio, Digital pathology, Tumor tissue composition

## Abstract

**Objectives:**

To evaluate signal enhancement ratio (SER) for tissue characterization and prognosis stratification in pancreatic adenocarcinoma (PDAC), with quantitative histopathological analysis (QHA) as the reference standard.

**Methods:**

This retrospective study included 277 PDAC patients who underwent multi-phase contrast-enhanced (CE) MRI and whole-slide imaging (WSI) from three centers (2015–2021). SER is defined as (SI_lt_ − SI_pre_)/(SI_ea_ − SI_pre_), where SI_pre_, SI_ea,_ and SI_lt_ represent the signal intensity of the tumor in pre-contrast, early-, and late post-contrast images, respectively. Deep-learning algorithms were implemented to quantify the stroma, epithelium, and lumen of PDAC on WSIs. Correlation, regression, and Bland-Altman analyses were utilized to investigate the associations between SER and QHA. The prognostic significance of SER on overall survival (OS) was evaluated using Cox regression analysis and Kaplan–Meier curves.

**Results:**

The internal dataset comprised 159 patients, which was further divided into training, validation, and internal test datasets (*n* = 60, 41, and 58, respectively). Sixty-five and 53 patients were included in two external test datasets. Excluding lumen, SER demonstrated significant correlations with stroma (*r* = 0.29–0.74, all *p* < 0.001) and epithelium (*r* = −0.23 to −0.71, all *p* < 0.001) across a wide post-injection time window (range, 25–300 s). Bland-Altman analysis revealed a small bias between SER and QHA for quantifying stroma/epithelium in individual training, validation (all within ± 2%), and three test datasets (all within ± 4%). Moreover, SER-predicted low stromal proportion was independently associated with worse OS (HR = 1.84 (1.17–2.91), *p* = 0.009) in training and validation datasets, which remained significant across three combined test datasets (HR = 1.73 (1.25–2.41), *p* = 0.001).

**Conclusion:**

SER of multi-phase CE-MRI allows for tissue characterization and prognosis stratification in PDAC.

**Clinical relevance statement:**

The signal enhancement ratio of multi-phase CE-MRI can serve as a novel imaging biomarker for characterizing tissue composition and holds the potential for improving patient stratification and therapy in PDAC.

**Key Points:**

- *Imaging biomarkers are needed to better characterize tumor tissue in pancreatic adenocarcinoma.*
- *Signal enhancement ratio* (*SER*)*-predicted stromal/epithelial proportion showed good agreement with histopathology measurements across three distinct centers.*
- *Signal enhancement ratio* (*SER*)*-predicted stromal proportion was demonstrated to be an independent prognostic factor for OS in PDAC.*

**Graphical Abstract:**

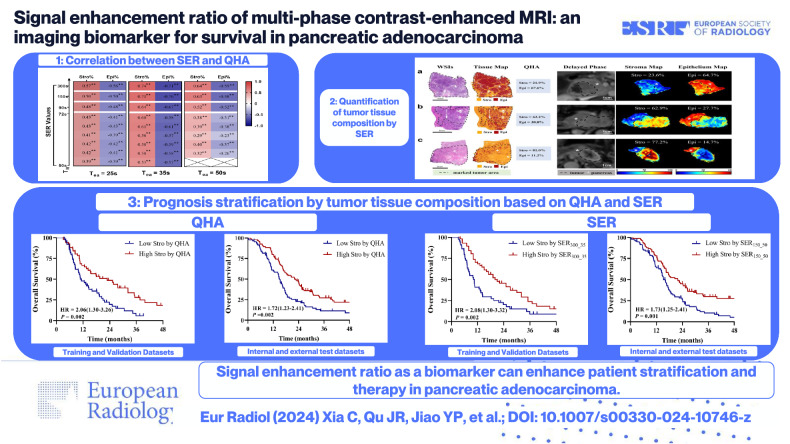

## Introduction

Pancreatic adenocarcinoma (PDAC) is one of the most lethal cancers with a dismal 5-year survival rate of 11% [1]. A hallmark feature of PDAC is its extensive desmoplastic stroma, which is thought to confer biological aggressiveness [2, 3]. It is reported that this dense stroma acts as a physical barrier to hinder the effective delivery of drugs [4, 5]. Moreover, the amount of stroma displays remarkable variability within the tumors as well as among patients, and this variability has been shown to yield prognostic information [6]. Generally, a low proportion of stroma predicts an unfavorable prognosis in PDAC [7–10]. However, current clinical imaging assessments predominantly rely on shape-based and volume-based descriptors [11]. There is a strong need to identify imaging biomarkers for characterizing tumor tissue composition in PDAC.

Multi-phase dynamic contrast-enhanced magnetic resonance imaging (DCE-MRI) is a highly sensitive modality for tumor characterization when compared to CT, owing to its superior soft tissue contrast and ability to provide functional information [12]. Quantitative parameters such as the volume transfer constant, *K*^trans^, have yielded some preliminary results in stroma estimation [13]. However, the technical complexities and relatively low image quality due to lower spatial resolution compared to conventional multi-phase contrast-enhanced MRI (CE-MRI), are major obstacles preventing the widespread use of quantitative parameters [14]. In contrast, signal enhancement ratio (SER) is a semiquantitative method to estimate signal intensity changes through a three-time-point examination [15]. Specifically, SER is defined as (*SI*_*lt*_ − *SI*_*pre*_)/(*SI*_*ea*_ − *SI*_*pre*_), where *SI*_*pre*_, *SI*_*ea*_, and *SI*_*lt*_ represent the signal intensity (SI) on pre-contrast, early post-contrast, and late post-contrast images, respectively. Previous studies have demonstrated close associations between SER and tumor biological behavior as well as prognostic outcomes in breast cancer [15, 16]. Nevertheless, few studies have investigated the potential of SER for PDAC tissue characterization.

With advances in digital pathology, Hao Fu et al proposed the first deep-convolutional neural network architecture for PDAC detection [17]. Bo Li et al employed a conditional generative adversarial model to segment tumors and stroma on whole-slide images (WSIs) [9]. These advancements facilitate an objective and standardized assessment of tumor tissue composition. However, to the best of our knowledge, the relationship between SER and quantitative tumor tissue composition has not been investigated in PDAC. Therefore, the objective of our study was to evaluate the potential of SER derived from multi-phase CE-MRI for characterizing tissue composition in PDAC, with quantitative histopathological analysis (QHA) as the reference standard. The prognostic value of SER was also analyzed.

## Methods

### Study patients

This retrospective study was approved by the institutional review board and the requirement for written informed consent was waived.

The study comprised consecutive patients with PDAC who underwent upfront surgery and preoperative multi-phase CE-MRI from three academic centers in China between 2015 and 2021. The internal dataset included patients who underwent either a 16-phase DCE-MRI or conventional multi-phase CE-MRI from Henan Cancer Hospital, Zhengzhou (Center A). Among patients with 16-phase DCE-MRI, a division was made into a training dataset (encompassing scans obtained 2015–2019) and a validation dataset (encompassing scans obtained 2020–2021). Patients who underwent conventional CE-MRI constituted an independent internal test dataset. Two external test datasets were included, consisting of patients who underwent conventional multi-phase CE-MRI at Xiangya Hospital of Central South University, Changsha (Center B), and Subei People’s Hospital, Yangzhou (Center C), respectively.

**Fig. 1:**
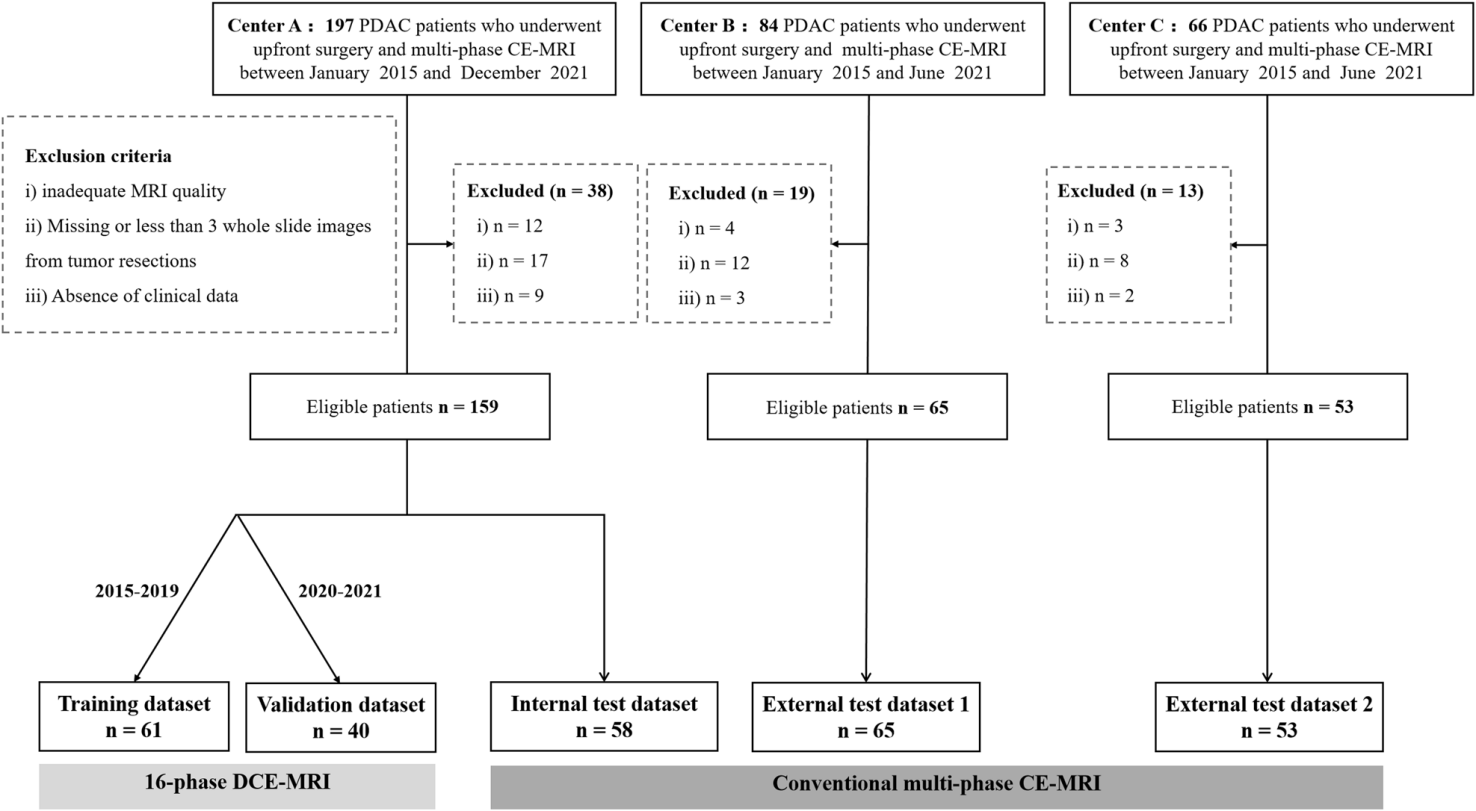
Flowchart of the patient selection process in training, validation, internal test, and two external test datasets. PDAC, pancreatic adenocarcinoma; DCE-MRI, dynamic contrast-enhanced MRI; WSIs, whole-slide images

The inclusion criteria for all patients were as follows: (i) upfront surgical resection with histopathological confirmation of PDAC, (ii) preoperative multi-phase CE-MRI within one month of surgery, and (iii) the availability of hematoxylin and eosin (H&E)-stained WSIs from tumor resections. The exclusion criteria were as follows: (i) inadequate MRI quality, (ii) missing or less than three WSIs, and (iii) absence of clinical data. The participant flowchart is shown in Fig. 1.

### MRI protocol and analysis

#### MRI protocol

The acquisition parameters for MRI are detailed in Appendix E1 and Table E1 and E2. In training and validation datasets, the MR sequences included 16-phase DCE-MRI, diffusion-weighted imaging (DWI), and T2-weighted imaging (T2WI). The acquisition of 16-phase DCE-MRI employed the CAIPIRINHA-Dixon-TWIST-VIBE technique. This technique involved capturing one pre-contrast phase (PRE), six consecutive arterial phases (AP) from 15 to 37 s after contrast injection, six consecutive portal venous phases (PVP) from 50 to 72 s, and followed by three delayed phases (DP) at 90, 150, and 300 s. For DWI, a single-shot echo-planar imaging pulse sequence was utilized with *b*-values of 50 and 800 s/mm^2^. Subsequently, apparent diffusion coefficient (ADC) maps were generated from the DWI scans of both *b*-values. In the internal and two external test datasets, conventional multi-phase CE-MRI was acquired with one PRE, AP (15–25 s), PVP (50–55 s), and DP (120–180 s).

#### Imaging preprocess and region of interest (ROI) measurements

For batch extraction of the region of interest (ROI) measurements, a grid and deformable registration technique via Elastix (https://elastix.lumc.nl/) was employed to achieve voxel-wise alignment for multi-phase CE-MRI images. Subsequently, ROIs for the tumor and paraspinal muscle were drawn by two radiologists (C.X. and Z.B., with 7 and 4 years of abdominal imaging experience, respectively), see Appendix E1 (electronic supplemental material) for more details of ROI delineations. The SER of the tumor at multi-phase CE-MRI was calculated as follows [18]:

$${{{{SER}}}}_{{{{lt}}}{{\_}}{{{ea}}}}=({{{{SI}}}}_{{{{lt}}}}-{{{{SI}}}}_{{{{pre}}}})/({{{{SI}}}}_{{{{ea}}}}-{{{{SI}}}}_{{{{pre}}}})$$

where $\,{{SI}}_{{pre}}$,$\,{{SI}}_{{ea}}$, and ${{SI}}_{{lt}}$ represent the signal intensity (SI) of the tumor ROI on pre-contrast, early post-contrast (AP or PVP: ranged from 15 to 72 s after contrast injection), and late post-contrast images (PVP or DP: ranged from 50 to 300 s), respectively. To assess the reproducibility of SER, two radiologists independently delineated the tumor ROIs on 16-phase DCE-MRI. Furthermore, in training and validation datasets, several additional parameters were acquired, including ADC values of the tumor, the tumor-to-muscle SI ratio on T2WI, and all pre- and post-contrast scans of 16-phase DCE-MRI.

#### SER analysis for quantifying tumor tissue composition

Given the wide post-injection time window of 16-phase DCE-MRI, we investigated the proper scanning timing of SER for characterizing tissue composition. Consistent with previous findings [13, 19], PDAC showed a rapid increase in enhancement during AP, followed by a gradual and slow increase in PVP and DP (Fig. S1). Consequently, for early post-contrast time point (*T*_ea_), we selected two clinically significant AP time points: The pancreatic parenchyma phase (approximately 35 s after contrast injection) is widely accepted as the optimal phase for detecting PDAC, while the late AP phase (approximately 25 s) is readily accessible and considered optimal for identifying liver tumors. Additionally, a commonly utilized PVP time point (approximately 50 s). Furthermore, for late post-contrast time points (*T*_lt_), we analyzed all time points in PVP and DP, ranging from 50 (excluding *T*_ea_ = 50) to 300 s, to investigate the impact of scan delay on tissue characterization. Spearman correlation analysis was employed to evaluate the correlation between SER and QHA. A time-*r*-value curve fitting analysis was combined to determine the optimal time points of *T*_ea_ and *T*_lt_ for characterizing tissue composition.

To assess the predictive capacity of SER for tissue quantification, the initial linear regression model was developed, using the SER at the optimally chosen *T*_ea_ and *T*_lt_ time points in training datasets. This model was subsequently validated in an entirely separate validation dataset. In instances where the optimally chosen time points were not acquired on conventional CE-MRI, a second linear regression model, utilizing the SER that demonstrated the highest correlation with QHA based on conventional CE-MRI scanning timings, was further constructed in the training dataset. This second model was then tested in three different test datasets to evaluate its clinical generalizability on conventional CE-MRI. The SER-fitted tissue composition maps were generated by employing the corresponding regression model and performing voxel-based calculations using Python scripts.

#### Quantitative histopathological analysis (QHA)

Three or more H&E-stained slides from tumor resection were selected for each patient and digitized at × 40 magnification at respective institutions. Two deep-learning-based segmentation models were developed to quantify tumor tissue composition on WSIs: one is for tumor detection and the other is for tissue segmentation. Image annotation of the “normal” and “tumor” region, as well as the “stroma”, “epithelium”, and “lumen” tissue was performed using ASAP (https://computationalpathologygroup.github.io/ASAP/), by two pathologists (Y.C.G. and M.Y.S. with 6 years of experience in gastrointestinal pathology).

##### Tumor detection

A VGG-19-based convolutional neural network was trained to recognize the normal and tumor regions. A total of 453 WSIs were annotated by pathologists to establish the ground truth for model training and validation. Of these, 209 WSIs were obtained from a publicly accessible cohort of The Cancer Genome Altas-Pancreatic Adenocarcinoma (TCGA-PAAD), available at https://www.cancer.gov. Then, 244 WSIs were selected in a randomized, center-balanced manner from the pool of WSIs at Centers A, B, and C. A total of 16,061 patches of size 512 × 512 pixels were extracted for model training and validation at a ratio of 7:3.

##### Tissue segmentation

A U-Net-based neural network was employed to segment stroma, epithelium, and lumen, as in our previous work [20]. A total of 391 WSIs from five TCGA cohorts, namely PAAD, breast cancer, lung adenocarcinoma, lung squamous cell carcinoma, and stomach adenocarcinoma, were manually annotated for model training. Ninety WSIs were selected in a randomized, center-balanced manner from Centers A, B, and C, and annotated for model-independent validation. A total of 17,159 patches of size 512 × 512 pixels were generated for model training and validation at a ratio of 7:3.

Finally, the proportion of stroma, epithelium, and lumen was calculated as the percentages of specific tissue area within the tumor area across all WSIs. The segmentation results were checked by two pathologists, and corrections were made as necessary.

### Statistical analysis

Statistical analyses were performed using SPSS (version 25.0, IBM; Armonk, NY, USA). Continuous and categorical variables were compared using the Kruskal–Wallis test and the Chi-square test or Fisher exact test, respectively. Interobserver agreements for SER were assessed using intraclass correlation coefficients (ICC). The agreements between SER and QHA for tissue quantification were evaluated through Bland-Altman plots and Pearson correlation tests. Survival analysis was performed on overall survival (OS), employing Kaplan–Meier curves with log-rank tests, and univariate- and multivariate Cox regression analyses. The cutoff point of stroma, epithelium, and SER for survival analysis was determined by their respective mean value. The significance level was set at *p* < 0.05, with a two-tailed approach for all analyses.

## Results

### Patient characteristics

A total of 277 patients were included in this study. The internal dataset comprised 159 patients from Center A, which was further divided into training, validation, and internal test datasets (*n* = 61, 40, 58, respectively). There were two external datasets from center B (*n* = 65) and center C (*n* = 53). The demographic and clinicalpathologic characteristics of the patients were largely balanced among the datasets (Table 1). Overall, 156 (56.3%) patients were male with a median age of 62 years (IQR, 54–68). The median intervals between MRI examinations and surgery were 3 (IQR, 2–6), 5 (4–7), 3 (2–6), 6 (3–8), and 6 (4–9) days for training, validation, internal test, and two external datasets, respectively.

**Table 1:** Clinicopathologic characteristics in training, validation, internal test, and two external datasets

Characteristics	Internal dataset (*n* = 159)			External datasets (*n* = 118)		*p-*value
	Training (*n* = 61)	Validation (*n* = 40)	Internal test (*n* = 58)	External test 1 (*n* = 65)	External test 2 (*n* = 53)	
Age (years)^a^	63 (56–68)	63 (56–70)	62 (53–66)	61 (52–67)	64 (54–71)	0.568
Sex (male)	34 (55.7)	21 (52.5)	32 (55.2)	37 (56.9)	32 (60.4)	0.958
CA 19-9 (> 37 U/mL)	47 (77.0)	28 (70.0)	44 (75.9)	47 (72.3)	37 (69.8)	0.875
Tumor location (head)	41 (67.2)	29 (72.5)	47 (81.0)	46 (70.8)	33 (62.3)	0.248
Tumor size (cm)^a^	3.5 (2.7–5.0)	3.5 (2.6–4.3)	3.5 (2.5–4.6)	3.0 (2.5–4.5)	2.8 (1.8–3.5)	0.004*
Pathological T stage						0.003*
T1	10 (16.4)	3 (7.5)	10 (17.2)	11 (16.9)	19 (35.9)	
T2	31 (50.8)	26 (65)	26 (44.8)	35 (53.9)	30 (56.6)	
T3	20 (32.8)	11 (27.5)	22 (37.9)	19 (29.2)	4 (7.5)	
Pathological N stage						0.256
N0	76 (75.2)	31 (77.5)	43 (74.1)	46 (70.8)	31 (58.5)	
N1 or N2	25 (24.8)	9 (22.5)	15 (25.9)	19 (29.2)	22 (41.5)	
Pathological TNM stage (AJCC 8th edition)						0.787
I	31 (50.8)	18 (45)	31 (53.4)	34 (52.3)	28 (52.8)	
II	27 (44.3)	20 (50)	27 (46.6)	28 (43.1)	22 (41.5)	
III	3 (4.9)	2 (5)	0 (0)	3 (4.6)	3 (5.7)	
Resection margin (R0)	58 (95.1)	39 (97.5)	46 (86.7)	62 (95.3)	48 (90.5)	0.650
Histological grade						0.880
Well or Moderate	48 (78.7)	33 (80.5)	48 (82.7)	51 (78.5)	45 (84.9)	
Poor	13 (21.3)	7 (17.5)	10 (17.2)	14 (21.5)	8 (15.1)	

*CA* carbohydrate antigen. * Indicates statistical significance; *p* < 0.05

^a^Continuous variables are presented with median and interquartile range and compared using the Kruskal–Wallis test; categorical variables are presented with numbers and percentages and compared using the Chi-square test or Fisher’s exact test

### Quantitative histopathological analysis

QHA was performed on 1027 WSIs from 277 (3.7 ± 0.8) patients in all datasets. The tumor detection model achieved a mean area under the receiver operating characteristic curve (AUC) of 0.94. The tissue segmentation model achieved mean Dice similarity coefficients for stroma, epithelium, and lumen were 0.89, 0.90, and 0.79, respectively (see Fig. S2 for visual examples).

The average proportion of stroma, epithelium, and lumen was 59.7 ± 13.7%, 25.5 ± 11.2%, and 5.4 ± 3.0% in PDAC. Poorly differentiated tumors exhibited a higher proportion of epithelium (poor, 36.2 ± 11.5% vs. well/moderate, 23.0 ± 9.6%; *p* < 0.001), and a lower proportion of stroma (poor, 48.8 ± 13.9% vs. well/moderate, 62.2 ± 12.4%; *p* < 0.001) than well or moderately differentiated PDACs, excluding lumen. Given the low proportion of lumen, our subsequent analysis focused on stroma and epithelium.

### Correlation analysis between QHA and MRI

For conventional MRI parameters, the tumor-to-muscle SI ratio on T2WI and all scans of 16-phase DCE-MRI showed negligible to significantly weak correlations with stroma (*r*, range −0.02 to 0.23) and epithelium (*r*, range −0.05 to −0.16); ADC showed weak yet significant correlations with stroma and epithelium (*r* = 0.32 and −0.29, respectively, both *p* < 0.001) in combined training and validation datasets.

**Fig. 2:**
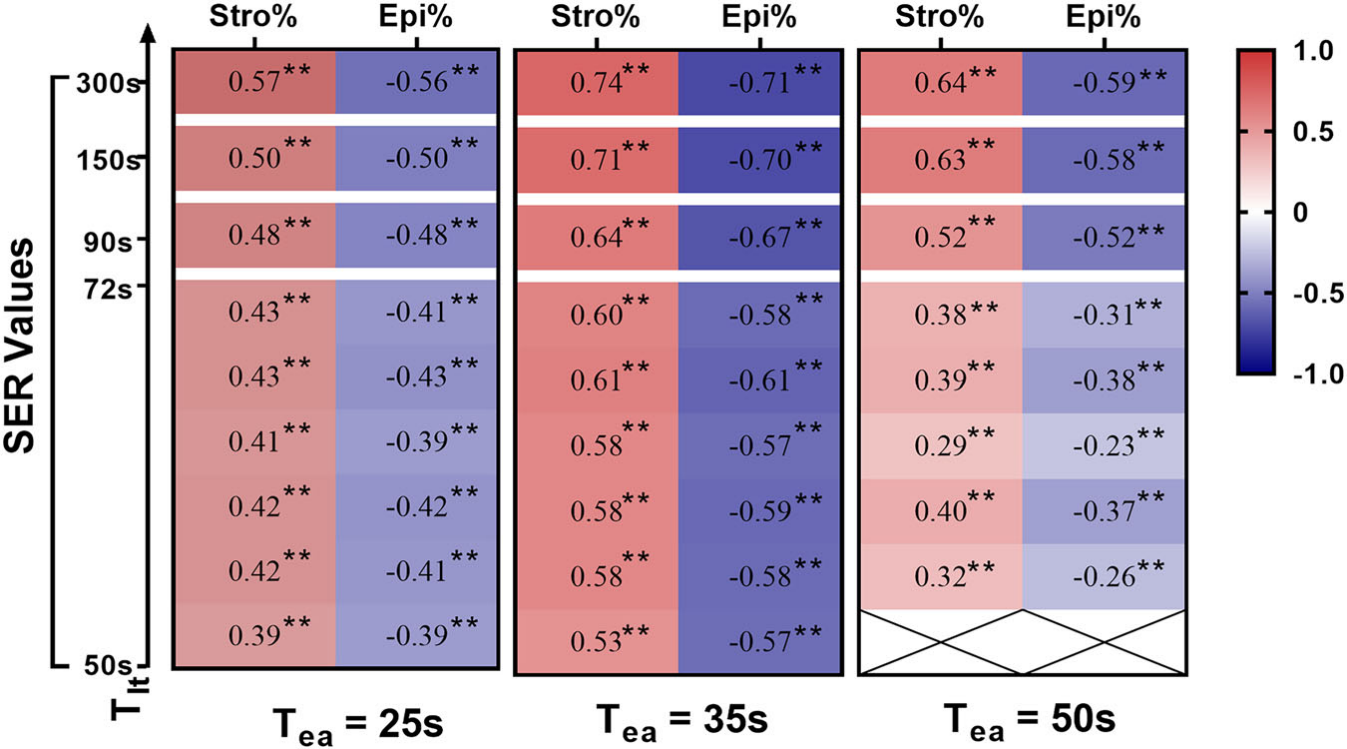
The SER and QHA correlation matrix in training and validation datasets. The Spearman correlation coefficients between SER and QHA measured the proportion of stroma (Stro%) and epithelium (Epi%) across various *T*_ea_ and *T*_lt_ time points (columns (*T*_ea_) represent the three early-contrast time points and rows (*T*_lt_) represent the late-contrast time points). SER, signal enhancement ratio; QHA, quantitative histopathological analysis. ** indicates *p* < 0.001

In contrast, Fig. 2 illustrates the significant correlations that exist between SER and both stroma (*r*, range 0.29–0.74, all *p* < 0.001) and epithelium (*r*, range −0.23 to −0.71, all *p* < 0.001) across a wide post-injection time window (range from 25 to 300 s). The highest correlations with both stroma and epithelium (*r* = 0.74 and −0.71, respectively) are demonstrated by SER_300_35_, thereby establishing the optimal SER. Further analysis from the time-*r*-value curve fitting suggests a proper *T*_lt_ time window of approximately 150–300 s (all |*r*| > 0.5). In accordance, the highest correlations are exhibited when *T*_ea_ = 35 (all |*r*| > 0.70), followed by *T*_ea_ = 50 (all |*r*| > 0.55) and *T*_ea_ = 25 s (all |*r*| > 0.50) (Fig. S3). For conventional CE-MRI scanning timing, the SER_150_50_ demonstrated the highest correlations with both stroma and epithelium (*r* = 0.63 and −0.58, respectively). The interobserver agreement for SER is good, with all ICC values exceeding 0.80 (range, 0.81–0.90).

### Linear regression analysis for tissue quantification

**Fig. 3:**
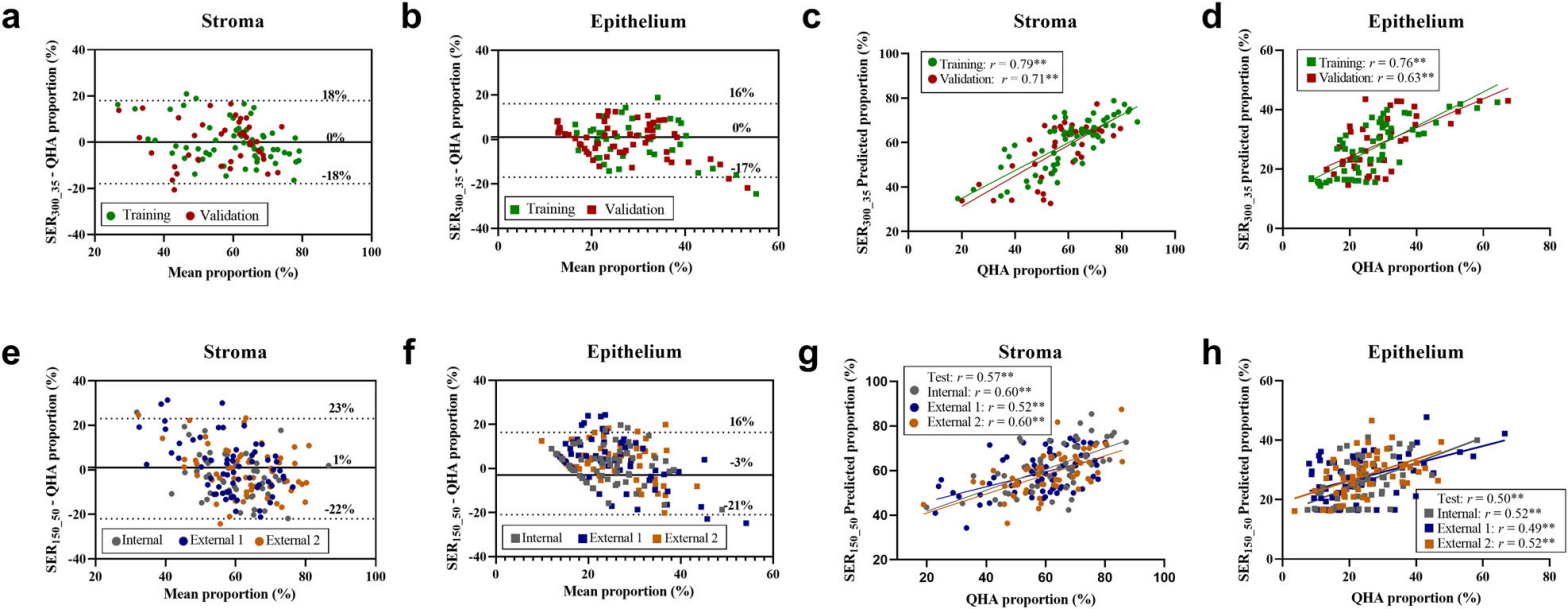
Bland-Altman plot and Pearson correlation plot for stroma and epithelium. **a**–**d** showing the agreement of stroma and epithelium quantified by using SER_300_35_ and QHA in training and validation datasets, respectively. **e**–**h** showing the agreement of stroma and epithelium quantified by using SER_150_50_ and QHA in the internal and two external test datasets. Bland-Altman plot is presented with a mean bias (bold line) and 95% limits of agreement (dot line). SER, signal enhancement ratio; QHA, quantitative histopathological analysis. ** indicates *p* < 0.001

**Fig. 4:**
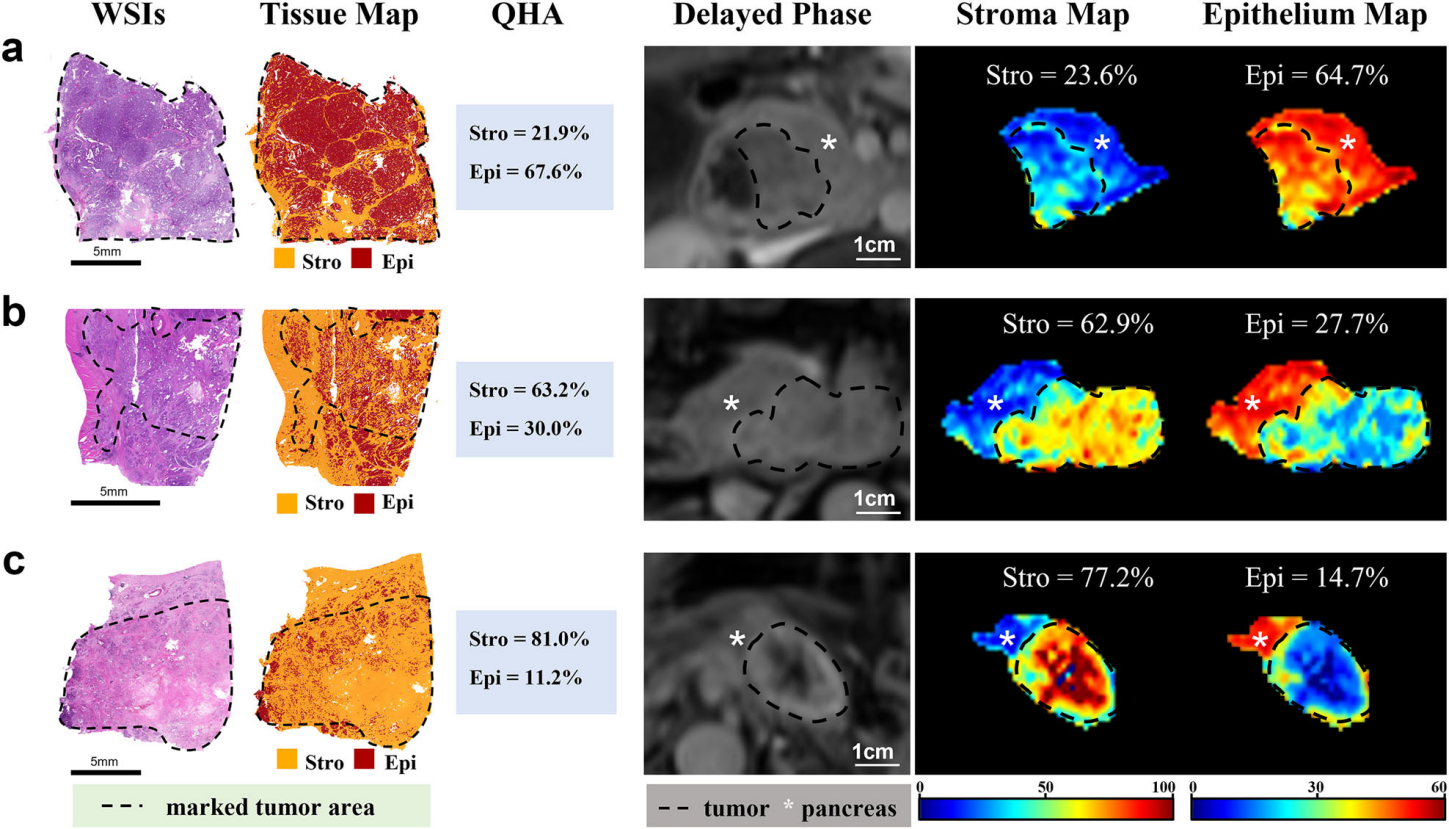
The SER-fitted maps for characterizing stroma and epithelium in PDAC. **a**–**c** Showing three representative tumors consisting of a low (high) (**a**), medium (medium) (**b**), and high (low) proportion of stroma (epithelium) (**c**), respectively. The SER-fitted stroma and epithelium maps provide a directly visualizable assessment of the proportion of stroma and epithelium in PDAC. SER, signal enhancement ratio; WSIs, whole-slide images; QHA, quantitative histopathological analysis; Stro, stroma; Epi, epithelium

To evaluate the predictive capacity of SER for tissue quantification, linear regression analyses were performed and the model development results are summarized in Appendix E2 and Table E3. In Bland-Altman analysis, bias and 95% limits of agreement (LA) indicated good accuracy for stroma/epithelium quantification by using the optimal SER_300_35_ model in both training and validation datasets (all mean bias within ±1% and 95% LA within ± 20%). Moreover, the low standard error (all < 10%) suggested a high level of precision (refer to Table E4 for details). Pearson correlation test showed strong correlations between SER_300_35_ and QHA in training and validation datasets for quantifying stroma (*r* = 0.79 vs. 0.71, both *p* < 0.001) and epithelium (*r* = 0.76 vs. 0.63, both *p* < 0.001) (Fig. 3). The visual examples of the SER-fitted stroma/epithelium maps are shown in Fig. 4.

To validate the clinical generalizability of SER on conventional CE-MRI, the second regression model, utilizing SER_150_50,_ was constructed and tested in the internal- and two external test datasets. Bland-Altman plots also showed a small bias between the measurements of SER_150_50_ and QHA in all three test datasets, with a mean bias of approximately ± 4% and 95% LA within ± 25% (see Table E4 for details). Pearson correlation test showed moderate correlations in three test datasets, for stroma (*r* = 0.60 vs. 0.52 vs. 0.60, all *p* < 0.001) and epithelium (*r* = 0.52 vs. 0.49 vs. 0.52, all *p* < 0.001) (Fig. 3).

### Univariable and multivariable analyses for prognostic significance

Univariate and multivariate Cox regression analyses were performed in combined training and validation datasets. The binary categorical marker of stromal proportion predicted by SER_300_35_ was an independent predictor of OS (cutoff = 60%, for low vs. high, HR = 1.84, 95% CI: 1.17–2.91, *p* = 0.009) after adjustment for the histologic grade, pathological T and N stage, and resection margin (Table 2). Similar results were obtained for the continuous marker of stromal proportion predicted by SER_300_35_ (HR = 0.98 (0.96–1.00), *p* = 0.019) (Table E5).

**Table 2:** Univariable and multivariable Cox regression analysis of the OS in combined training and validation datasets

Parameters	Univariate		Multivariate	
	Hazard ratio	*p-*value	Hazard ratio	*p*-value
Age (> 65 vs. ≤ 65 years)	0.90 (0.58–1.39)	0.626		
Sex (male vs. female)	0.86 (0.56–1.33)	0.497		
CA19-9 level (> 37 vs. ≤ 37 U/mL)	1.15 (0.70–1.89)	0.574		
Tumor location (head vs. body/tail)	1.05 (0.66-1.69)	0.834		
Pathological T stage (T3 vs. T1-2)	1.38 (0.87–2.18)	0.17	1.10 (0.67–1.79)	0.712
Pathological N stage (N1-2 vs. N0)	1.79 (1.10–2.91)	0.019*	1.79 (1.10–2.93)	0.02*
Histological grade (poor vs. well/moderate)	3.07 (1.80–5.24)	< 0.001**	3.06 (1.73–5.41)	< 0.001**
Resection margin (R1 vs. R0)	3.14 (1.12–8.27)	0.03*	1.69 (0.55–5.13)	0.358
LVI (positive vs. negative)	1.27 (0.75–2.15)	0.379		
Perineural invasion (positive vs. negative)	0.99 (0.62–1.57)	0.963		
SER_300_35_ (low vs. high)	1.98 (1.28–3.07)	0.002*	...	...
Stro% predicted by SER_300_35_ (low vs. high)	1.96 (1.27–3.04)	0.003*	1.84 (1.17–2.91)	0.009*
Epi% predicted by SER_300_35_ (low vs. high)	0.64 (0.42–0.99)	0.049*	...	...
Adjuvant chemotherapy (yes vs. no)	0.75 (0.48–1.17)	0.211		

Variables with *p* < 0.10 in the univariate analysis were included in the multivariate analysis. Data in parentheses are 95% confidence intervals

*OS* overall survival, *LVI* lymphovascular invasion, *SER* signal enhancement ratio, *Stro%* the proportion of stroma, *Epi%* the proportion of epithelium

* indicates *p* < 0.05, ** indicates *p* < 0.001

**Fig. 5:**
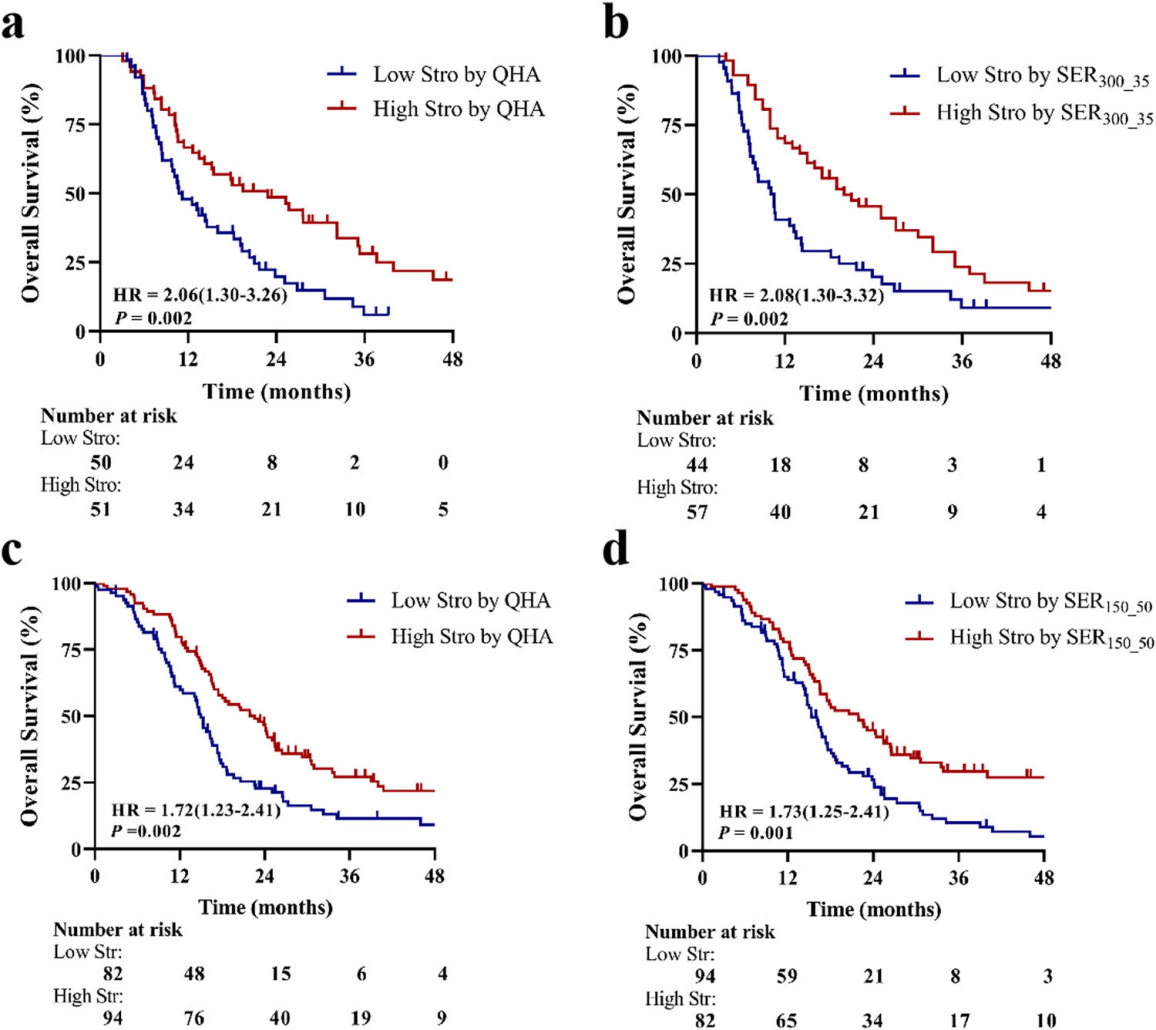
The prognostic value of stroma quantified by QHA and SER. Kaplan–Meier curves for overall survival (OS) according to the stromal proportion quantified by QHA (**a**), and SER_300_35_ (**b**) across the training and validation datasets, as well as QHA (**c**), and SER_150_50_ (**d**) across the internal and two external test datasets. Kaplan–Meier curves show that a low stromal proportion is significantly associated with worse OS in PDAC. QHA, quantitative histopathological analysis; SER, signal enhancement ratio; Stro, stroma

Kaplan–Meier curves demonstrated that the stromal proportion predicted by SER_300_35_ identified substantially different OS periods between two patient subgroups (median OS: low 10.1 vs. high 20.3 months, HR = 2.08 (1.30–3.32), *p* = 0.002). Notably, these findings were comparable to those obtained through QHA (low 10.7 vs. high 22.8 months, HR = 2.06 (1.30–3.26), *p*  = 0.002). Similar findings were observed in the stromal proportion predicted by SER_150_50_ across three combined test datasets (low 15.3 vs. high 20.6 months, HR = 1.73 (1.25–2.41), *p* = 0.001), compared to QHA (low 15.0 vs. high 21.9 months, HR = 1.72 (1.23–2.41), *p*  = 0.002) (Fig. 5).

## Discussion

Characterization of tumor tissue composition has shown promise for enhancing patient stratification and therapy in PDAC [3, 4, 21]. In this study, significant correlations were observed between SER and QHA-measured stroma/epithelium in PDAC. The high interobserver reproducibility of SER, coupled with its demonstrated concordance in tissue quantification with QHA across three distinct centers, reinforces its reliability and generalizability as a potential imaging biomarker for characterizing tissue composition in PDAC. Moreover, our analysis demonstrated that SER-predicted a low stromal proportion was independently associated with worse OS in training and validation datasets, and this association retained its significant prognostic value across three combined test datasets. These findings underscore the potential of SER for tissue characterization and prognostic stratification in PDAC.

The visual assessment of quantitative histopathological features is prone to interobserver variability, and thus we have developed deep-learning algorithms for QHA. Compared to previous studies [9, 17], our segmentation models have exhibited comparable performance in a relatively large multicenter dataset. Moreover, the stromal proportion measured by QHA is in line with prior studies by using immunohistochemical staining [22, 23], indicating the good accuracy of our segmentation models.

To investigate the optimal time points of SER for tissue characterization, our study utilized a 16-phase DCE-MRI, which offers a wider post-injection time window compared to conventional CE-MRI for PDAC characterization. Our investigation revealed that the optimal tissue characterization using SER may be achieved by combining *T*_ea_ = 35 and *T*_lt_ = 300 s. These findings align with prior studies that have demonstrated a slow and gradual enhancement pattern in PDAC, with PPP providing the maximum contrast for PDAC detection [13, 19].

SER of multi-phase CE-MRI can provide a semiquantitative approximation of the redistribution rate constant [15, 18]. The correlation between SER and QHA may potentially be elucidated by different tissue contrast agent concentrations. Specifically, the stroma, which is comprised of dense fibrotic tissue, increases the volume of distribution for the contrast agent. Conversely, gadolinium is incapable of permeating intact cell membranes within the epithelium [14].

To the best of our knowledge, this is the first study to investigate SER as a potential imaging biomarker for quantifying tissue composition in PDAC. To assess the predictive capacity of SER, we developed a first linear regression model utilizing the optimal SER_300_35_. Our results demonstrated that the stromal/epithelial proportion predicted by SER_300_35_ exhibited a high level of agreement with the measurements of QHA, thereby supporting the efficacy of SER for tissue quantification. Furthermore, recognizing that SER may be influenced by varying scanning systems [18], we conducted additional validation using conventional CE-MRI data from three distinct centers. Despite the optimal time points not routinely captured in conventional CE-MRI [24–26], the stromal/epithelial proportion predicted by SER_150_50_ still demonstrated a good agreement with the measurements of QHA. This finding further emphasizes the applicability and generalizability of SER for tissue quantification in clinical practice.

A majority of studies have shown that PDAC with a low proportion of stroma tends to exhibit worse histological grades and poorer outcomes [8–10, 27, 28], which aligns with our findings. However, there exists conflicting evidence in the literature, suggesting that a low proportion of stroma predicts longer survival [23, 29]. This contradiction can partly be attributed to the limitations of small sample sizes and discrepancies in stroma quantification methods. Notably, the conflicting studies predominantly rely on subjective and random regional assessments or solely evaluate stroma in the most severe areas, both of which fail to adequately represent the entire tumor due to the spatial heterogeneity of PDAC [30]. In contrast, QHA enables an objective and standardized approach to analyze WSIs in multicenter cohorts. Our findings demonstrated that the stromal proportion predicted by SER achieved comparable performance to QHA in stratifying patients. Furthermore, when subjected to multivariate analysis, the SER-predicted stromal proportion remained an independent predictor in training and validation datasets, and demonstrated significant prognostic value across three test datasets. These findings might have significant implications for PDAC management.

## Limitations

We acknowledge several limitations in our study. First, the retrospective design introduces potential bias in patient selection. Second, the sample size was relatively small after separating training, validation, and internal and external test datasets. Third, the retrospective nature of the study prevented us from conducting spatial registration between WSIs and MRI data. Furthermore, future studies should be conducted with prospective cohorts, preferably in a multicenter setting.

## Conclusions

In conclusion, our study demonstrated that SER of multi-phase CE-MRI is a potential imaging biomarker for tissue composition characterization and prognosis stratification in PDAC.

## Supplementary information

ELECTRONIC SUPPLEMENTARY MATERIAL

## Abbreviations

AP: Arterial phases
DCE-MRI: Dynamic contrast-enhanced magnetic resonance imaging
DP: Delayed phases
PDAC: Pancreatic adenocarcinoma
PVP: Portal venous phases
QHA: Quantitative histopathological analysis
ROI: Region of interest
SER: Signal enhancement ratio
WSI: Whole-slide image
