# Supplementary material for: Signal enhancement ratio of multi-phase contrast-enhanced MRI: an imaging biomarker for survival in pancreatic adenocarcinoma
Source: Eur Radiol. 2024 May 15;34(11):7460–70. doi: 10.1007/s00330-024-10746-z (PMC11519106; doi:10.1007/s00330-024-10746-z)
Supplement: Supplementary file 1 — ELECTRONIC SUPPLEMENTARY MATERIAL [file 330_2024_10746_MOESM1_ESM.pdf]

Signal enhancement ratio of multi-phase contrast-enhanced MRI: an imaging biomarker for survival in pancreatic adenocarcinoma

ELECTRONIC SUPPLEMENTARY MATERIAL

Appendix E1. MRI protocol and analysis

I) MRI protocol

The parameters of 16-phase DCE-MRI in training and validation datasets and conventional multi-phase CE-MRI in the internal and two external test datasets are listed in Tables E1 and E2, respectively. In training and validation datasets, T2WI was obtained using a 2D turbo spin echo sequence using acquisition matrix = 240 × 320, FOV = 285 mm × 380 mm, slice thickness = 4 mm, TE = 78 ms, TR = 6765 ms. Diffusion-weighted imaging (DWI) was obtained with acquisition matrix = 108 × 136, FOV= 285 mm × 360 mm, slice thickness = 4 mm, TE = 51 ms, TR = 2200 ms, b-values = 50, and 800 s/mm^2.

II) Regions of Interest (ROIs) delineation and analysis

Two radiologists (C.X. and Z.B.) independently performed ROI delineation by using a software tool "ITK-SNAP"(www.itk-snap.org), who were blinded to clinical and histopathological data. The three-dimensional ROIs of tumors were drawn on axial images of DCE-MRI in all datasets, and on axial T2WI and DWI in training and validation datasets. The cystic or necrotic areas were carefully excluded. The two-dimensional ROIs (100-150 mm^2) of paraspinal muscle were placed on both sides at

the renal hilum level. The tumor-to-muscle SI ratio was calculated as the signal intensity of the tumor to paraspinal muscle on T2WI and all pre- and post-contrast scans on 16-phase DCE-MRI.

Appendix E2. Linear regression analysis for tissue quantification

To evaluate the predictive capacity of SER for tissue quantification, we first integrate the optimal SER_{300_35} into a linear or piece-wise linear regression model utilizing the training dataset. Second, to evaluate the generalizability of SER for tissue quantification on conventional multi-phase CE-MRI, we integrate the SER_{150_50} into a linear or piece-wise linear regression model, again employing the training dataset.

The performance of the models in the validation dataset is summarized in Table E3. The piece-wise linear regression models show better performance than the linear models, as indicated by Mean Absolute Error (MAE) and Root Mean Squared Error (RMSE). As a result, the definitive model selected is a piece-wise linear regression model, incorporating the SER_{300_35} or SER_{150_50} value.:

$$\text{Stromal proportion } (x=SER_{300\_35}):\ y = \begin{cases} 59.8x - 18.7, & (x < 1.36) \\ 12.4x + 45.6, & (x \geq 1.36) \end{cases}$$

$$\text{Epithelial proportion } (x=SER_{300\_35}):\ y = \begin{cases} -29.3x + 68.7, & (x < 1.77) \\ -2.94x + 22.2, & (x \geq 1.77) \end{cases}$$

$$\text{Stromal proportion } (x=SER_{150\_50}):\ y = \begin{cases} 68.5x - 18.2, & (x < 1.29) \\ 34.5x + 25.5, & (x \geq 1.29) \end{cases}$$

$$\text{Epithelial proportion } (x=SER_{150\_50}):\ y = \begin{cases} -58.6x + 93.3, & (x < 1.31) \\ -0.86x + 17.7, & (x \geq 1.31) \end{cases}$$

Table E1. Parameters of 16-phase DCE-MRI in training and validation datasets

Parameter	
Magnetic field strength	3.0 T
Sequence	CAIPIRINHA-Dixon-TWIST-VIBE
Manufacturer	SIEMENS
TR/TE, ms	4.25/1.28
Flip angle, degree	12
Matrix size	320 × 210
Slice thickness (mm)	3
Pixel spacing (mm)	1.3 × 1.3
Field of View (mm)	400 × 263
Parallel imaging	CAIPIRINHA: 2 × 2-1
Fat suppression	2-point Dixon method
TWIST (A/B)	20%/25%
Dynamic reconstruction mode	Forward share
Temporal resolution, s	2.64
Contrast agent	Gd-DOPA
Contrast dose (mmol/kg)	0.1
Injection rate (ml/s)	2.5

Note. CAIPIRINHA = controlled aliasing in parallel imaging results in higher acceleration, TWIST = time-resolved imaging with interleaved stochastic trajectories, VIBE = volumetric interpolated breath-hold examination.

Table E2. parameters of conventional multi-phase CE-MRI in internal and two external test datasets

Parameters	Internal test dataset	External test dataset 1	External test dataset 2
Magnetic field strength	3.0 T	1.5 T	3.0 T
Sequence	LAVA	VIBE	LAVA
Manufacturer	GE Medical Systems	SIEMENS	GE Medical Systems
TR/TE (ms)	2.67/1.24	4.49/2.19	4.25/1.67
Flip angle (degree)	12	10	15
Matrix size	512 × 512	320 × 260	512 × 512
Slice thickness (mm)	4	3	5
Pixel spacing (mm)	0.7 × 0.7	1.2 × 1.2	0.8 × 0.8
Field of View (mm)	380 × 380	380 × 308	400 × 400
Contrast agent	Gd-DOPA	Gd-DTPA	Gd-DTPA/Gadodiamide
Contrast dose (mmol/kg)	0.1	0.1	0.1
Injection rate (ml/s)	2.5	2.5	2.5
Acquisition time	AP: 15-25 s; PVP: 50s; DP: 150-180s	AP: 15-25s; PVP: 55s; DP: 180s	AP: 15s; PVP: 50s; DP: 120-150s

Note. VIBE = volumetric interpolated breath-hold examination, LAVA = liver acceleration volume acquisition, TR = repetition time, TE = echo time, AP = arterial phase, PVP = portal venous phase, DP = delayed phase.

Table E3. Model performances for stroma or epithelium proportion regression in the validation dataset.

Model(independent variables)	Stroma(%)		Epithelium(%)	
	MAE	RMSE	MAE	RMSE
Piecewise linear regression (SER_{300_35})	8.0	9.6	7.3	9.2
Linear regression (SER_{300_35})	8.1	10.2	7.6	9.7
Piecewise linear regression (SER_{150_50})	9.3	12.7	8.5	11.5
Linear regression (SER_{150_50})	9.4	12.8	8.5	11.5

Note. MAE = Mean Absolute Error; RMSE = Root Mean Squared Error.

Table E4. Bias and Agreement of Bland-Altman Analysis between SER and QHA for quantifying stroma and epithelium

	Stroma (%)			Epithelium (%)		
	**Bias**	**SD**	**LA**	**Bias**	**SD**	**LA**
SER_{300_35}						
Training dataset	0	8.9	-17.4 to 17.4	-0.3	8.3	-16.6 to 15.9
Validation dataset	-0.1	9.7	-19.1 to 18.9	-0.8	9.3	-19.0 to 17.5
SER_{150_50}						
Training dataset	0	11.5	-22.6 to 22.6	0	9.4	-18.4 to 18.4
Validation dataset	-1.7	10.3	-21.8 to 18.4	1.1	10.0	-18.5 to 20.7
Internal test dataset	-0.9	11.1	-22.6 to 20.9	1.9	8.5	-14.7 to 18.4
External test dataset 1	1.25	12.2	-22.7 to 25.2	3.5	11.1	-18.2 to 25.1
External test dataset 2	-2.3	10.6	-23.3 to 18.1	3.5	8.9	-13.8 to 20.0

Note. SD = standard deviation, LA = 95% limits of agreement.

Table E5. Univariable and multivariable Cox regression analysis of the OS in combined training and validation datasets

Parameters	Univariate		Multivariate	
	Hazard Ratio	*p*-value	Hazard Ratio	*p*-value
Age (>65 vs. ≤ 65 y)	0.90 (0.58-1.39)	0.626		
Sex (male vs. female)	0.86 (0.56-1.33)	0.497		
CA19-9 level (>37 vs. ≤ 37 U/ml)	1.15 (0.70-1.89)	0.574		
Tumor location (head vs. body/tail)	1.05 (0.66-1.69)	0.834		
Pathological T stage (T3 vs. T1-2)	1.38 (0.87-2.18)	0.17	1.24 (0.76-2.02)	0.394
Pathological N stage (N1-2 vs. N0)	1.79 (1.10-2.91)	0.019*	1.72 (1.04-2.82)	0.033*
Histological grade (poor vs. well/moderate)	3.07 (1.80-5.24)	<0.001**	2.92 (1.64-5.19)	<0.001**
Resection margin (R1 vs. R0)	3.14 (1.12-8.27)	0.03*	1.54 (0.49-4.84)	0.464
LVI (positive vs. negative)	1.27 (0.75-2.15)	0.379		
Perineural invasion (positive vs. negative)	0.99 (0.62-1.57)	0.963		
SER_{300_35}	0.50 (0.29-0.86)	0.013*	...	...
Stro% predicted by SER_{300_35}	0.97 (0.96-0.99)	0.003*	0.98 (0.96-1.00)	0.019*
Epi% predicted by SER_{300_35}	1.04 (1.01-1.06)	0.004*	...	...
Adjuvant chemotherapy (yes vs. no)	0.75 (0.48-1.17)	0.211		

Note. Variables with $P < 0.10$ in the univariate analysis were included in the multivariate analysis. Data in parentheses are 95% confidence intervals. OS = overall survival, LVI= lymphovascular invasion, SER = Signal Enhancement Ratio, Stro% = the proportion of stroma, Epi% = the proportion of epithelium. * indicates $P < 0.05$, ** indicates $P < 0.001$.

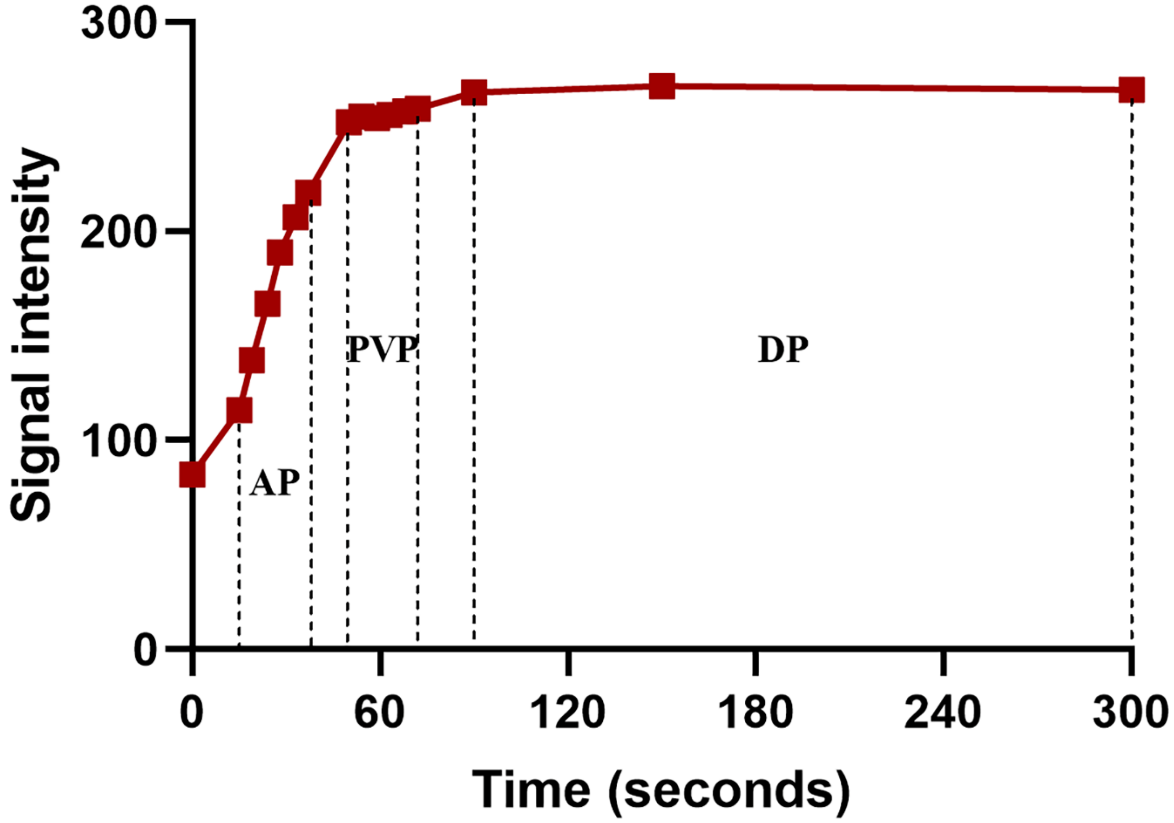

Fig S1. Time-signal intensity curve of pooled pancreatic adenocarcinoma.
AP, arterial phases; PVP, portal venous phases; DP, delayed phases.

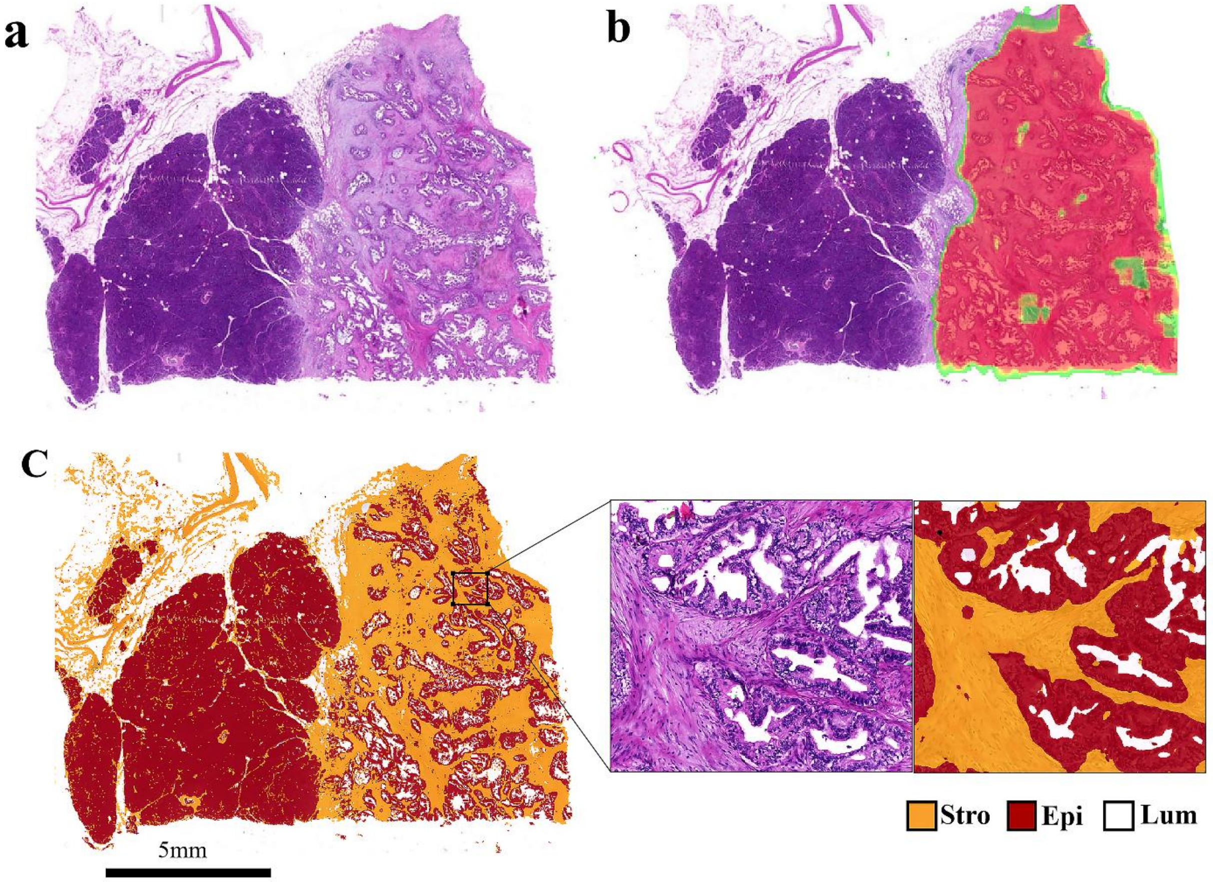

Fig S2. Deep learning models for automatic tumor detection and tissue segmentation.
(a)whole slide image, **(b)** tumor heatmap, where red represents high tumor likelihood, transparency and green indicate low tumor likelihood, **(c)** segmented tissue map.
Stro = stroma, Epi = epithelium, Lum = lumen.

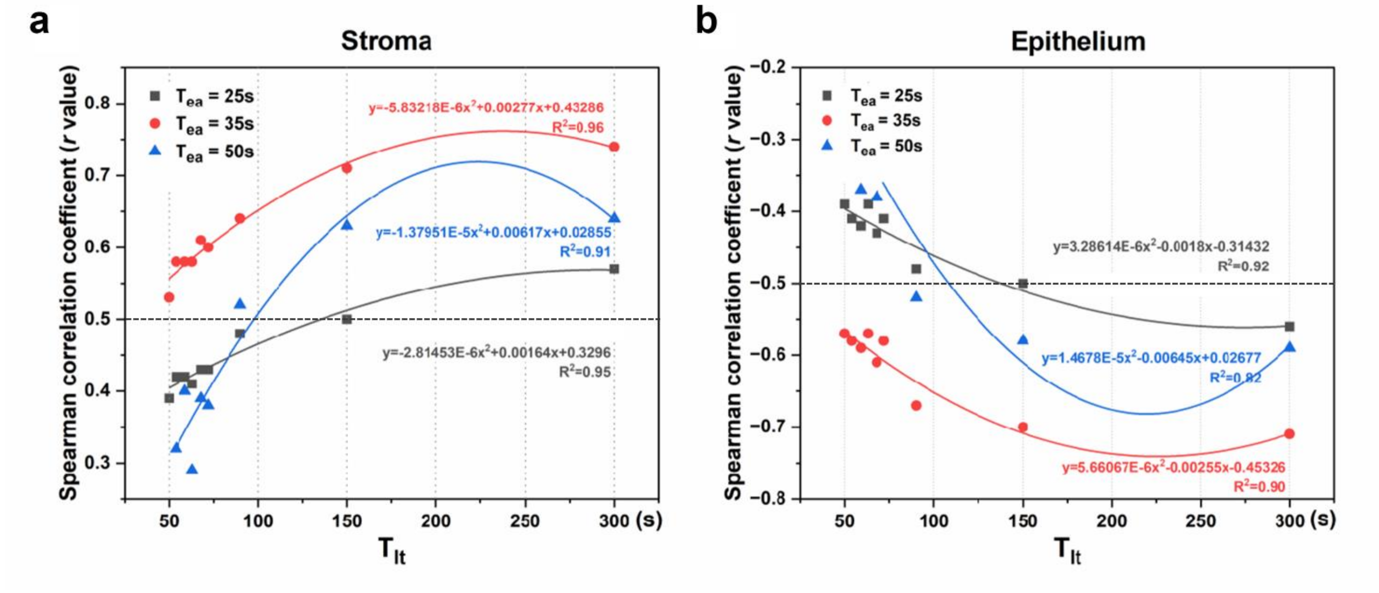

Fig S3. Time - *r* value curve fitting analysis.

a, b showing the Spearman *r* value between SER and QHA measured stroma **(a)**, and epithelium **(b)** across various T_{ea} and T_{lt} time points. Polynomial curve fitting analysis shows the proper T_{lt} time window is around 150 to 300 seconds after contrast injection (all | r | > 0.5).

T_{ea} = the early-contrast time point, T_{lt} = the late-contrast time point, SER = Signal Enhancement Ratio.
